# Adaptation of *Rhizobium leguminosarum *to pea, alfalfa and sugar beet rhizospheres investigated by comparative transcriptomics

**DOI:** 10.1186/gb-2011-12-10-r106

**Published:** 2011-10-21

**Authors:** Vinoy K Ramachandran, Alison K East, Ramakrishnan Karunakaran, J Allan Downie, Philip S Poole

**Affiliations:** 1 School of Biological Sciences, University of Reading, Reading, RG6 6AJ, UK; 2 Department of Molecular Microbiology, John Innes Centre, Norwich Research Park, Norwich, NR4 7UH, UK

## Abstract

**Background:**

The rhizosphere is the microbe-rich zone around plant roots and is a key determinant of the biosphere's productivity. Comparative transcriptomics was used to investigate general and plant-specific adaptations during rhizosphere colonization. *Rhizobium leguminosarum *biovar *viciae *was grown in the rhizospheres of pea (its legume nodulation host), alfalfa (a non-host legume) and sugar beet (non-legume). Gene expression data were compared to metabolic and transportome maps to understand adaptation to the rhizosphere.

**Results:**

Carbon metabolism was dominated by organic acids, with a strong bias towards aromatic amino acids, C1 and C2 compounds. This was confirmed by induction of the glyoxylate cycle required for C2 metabolism and gluconeogenesis in all rhizospheres. Gluconeogenesis is repressed in *R. leguminosarum *by sugars, suggesting that although numerous sugar and putative complex carbohydrate transport systems are induced in the rhizosphere, they are less important carbon sources than organic acids. A common core of rhizosphere-induced genes was identified, of which 66% are of unknown function. Many genes were induced in the rhizosphere of the legumes, but not sugar beet, and several were plant specific. The plasmid pRL8 can be considered pea rhizosphere specific, enabling adaptation of *R. leguminosarum *to its host. Mutation of many of the up-regulated genes reduced competitiveness for pea rhizosphere colonization, while two genes specifically up-regulated in the pea rhizosphere reduced colonization of the pea but not alfalfa rhizosphere.

**Conclusions:**

Comparative transcriptome analysis has enabled differentiation between factors conserved across plants for rhizosphere colonization as well as identification of exquisite specific adaptation to host plants.

## Background

Interactions between micro-organisms and plant roots in the rhizosphere are a key determinant of plant productivity. There is a two-way dialogue in which plants manipulate the rhizosphere's microbial community, which, in turn, profoundly alters plant growth [1]. Plants exude up to 11% of fixed carbon via their roots, including both small organic compounds and those that act as signaling molecules [2]. Carbon export on this scale must have a significant impact on rhizosphere micro-organisms, leading to alterations in community structure and function. The rhizosphere is an environment in which there are co-evolved mutualistic relationships between plants and microbes [1]. The best characterized beneficial associations are mutualisms with *Rhizobium *and mycorrhizae, but many other bacteria promote plant growth [1].

The symbiosis between rhizobia and legume hosts has been studied in great detail because their reduction of atmospheric N_2 _to ammonium is one of the largest inputs of available nitrogen into the biosphere [3]. Colonization of legume roots by rhizobia induces development of root nodules; in most studied systems, plant-released flavonoids induce rhizobia to synthesize lipochitooligosaccharide Nod factors, which induce root hair deformation and nodule morphogenesis [3]. Rhizobia are entrapped by curling root hairs and induce the plant to form infection threads that grow through the root hair and root cortical cells, leading to nodule formation. Bacteria are released from infection threads by endocytosis and surrounded by a plant membrane that controls exchange of carbon and nitrogen between the plant cytosol and rhizobia [4]. Despite detailed knowledge of root hair infection and nodule formation in legumes, little is known about the critical steps of rhizosphere colonization. By comparing *Rhizobium leguminosarum *colonization of the rhizosphere of its host legume with that of a non-host legume and a non-legume, we have been able, for the first time, to draw general conclusions about life in the plant rhizosphere as well as examine specific adaptation to a legume host.

## Results and discussion

Rhizobia provide a special advantage when studying the plant rhizosphere as bacterial responses can be investigated during colonization of the rhizosphere of a specific host legume (for example, pea), a non-host legume (alfalfa) and a non-legume (sugar beet). In addition, we are able to chart metabolic activity in the rhizosphere by comparison to the *Sinorhizobium meliloti *transportome, which comprises a large induction map for 76 identified ATP-binding cassette (ABC) and tripartite ATP-independent periplasmic (TRAP) transport systems in rhizobia [5]. This induction map was extended in this study with a series of microarrays of free-living cultures grown on a variety of metabolites (Table 1).

**Table 1:** Microarray experiments performed with *R.leguminosarum *biovar *viciae *Rlv3841

Array Express accession number^a^	Condition 1	Condition 2	Biological replicates	Results shown
E-MEXP-2844	Pyruvate/NH_4_^+^/hesperetin (1 μM)	Pyruvate/NH_4_^+^	3	Additional file 6
E-MEXP-2846	Pea root exudate	Pyruvate/NH_4_^+^	3	Additional file 6
E-MEXP-2845	21 day pea rhizosphere 1 dpi	Glucose/NH_4_^+^	4	Additional file 6
E-MEXP-2845	14 day pea rhizosphere 1 dpi	Glucose/NH_4_^+^	4	Additional file 6
E-MEXP-2845, E-MEXP-2848	7 day pea rhizosphere 1 dpi	Glucose/NH_4_^+^	5	Additional file 6
E-MEXP-2848	7 day pea rhizosphere 3 dpi	Glucose/NH_4_^+^	5	Additional file 6
E-MEXP-2848, E-MEXP-2852, E-MEXP-2854	7 day pea rhizosphere 7 dpi	Glucose/NH_4_^+^	5	Additional file 7
E-MEXP-2852	7 day alfalfa rhizosphere 7 dpi	Glucose/NH_4_^+^	3	Additional file 7
E-MEXP-2852	7 day sugar beet rhizosphere 7 dpi	Glucose/NH_4_^+^	3	Additional file 7
E-MEXP-2849	7 day pea rhizosphere 7 dpi	7 day alfalfa rhizosphere 7 dpi	4	Additional file 6
E-MEXP-2849	7 day pea rhizosphere 7 dpi	7 day sugar beet rhizosphere 7 dpi	4	Additional file 6
E-MEXP-2849	7 day alfalfa rhizosphere 7 dpi	7 day sugar beet rhizosphere 7 dpi	4	Additional file 6
E-MEXP-2854	7 day pea rhizosphere 7 dpi inoculated with 10^3 ^CFU	Glucose/NH_4_^+^	3	Additional file 6
E-MEXP-2857	Formate/pyruvate	Pyruvate/NH_4_^+^	1	Additional file 9
E-MEXP-2857	Protocatechuate	Pyruvate/NH_4_^+^	1	Additional file 9
E-MEXP-2857	4-Hydroxybenzoate	Pyruvate/NH_4_^+^	1	Additional file 9
E-MEXP-2857	Phenylalanine/pyruvate/NH_4_^+^	Pyruvate/NH_4_^+^	1	Additional file 9
E-MEXP-2857	Proline/pyruvate	Pyruvate/NH_4_^+^	1	Additional file 9
E-MEXP-2857	N-limited (glucose/1 mM NH_4_^+^)	Glucose/10 mM NH_4_^+^	1	Additional file 9
E-MEXP-2857	L-Arabinose/NH_4_^+^	Glucose/NH_4_^+^	1	Additional file 9
E-MEXP-2857	Galactose/NH_4_^+^	Glucose/NH_4_^+^	1	Additional file 9
E-MEXP-2857	Arabinogalactan/pyruvate/NH_4_^+^	Pyruvate/NH_4_^+^	1	Additional file 9

Microarrays comparing growth on acetate, acetoacetate, inositol, succinate, glucose and pyruvate as carbon sources have been published previously [8]. All carbon sources were 10 mM, except pyruvate (30 mM), protocatechuate (3 mM), 4-hydroxybenzoate (3 mM), phenylalanine (5 mM), formate (40 mM) and arabinogalactan (10 mg/ml). ^a^Where two or more accession numbers are given they form part of different time courses. Inoculation of all rhizospheres was performed with 10^8 ^CFU unless otherwise stated.

At the start of this study three variables were compared: (i) length of incubation of bacteria in the rhizosphere (bacteria harvested at 1, 3 and 7 days post-inoculation (dpi) of 7-day-old pea plants (Table 1; Additional file 1)); (ii) age of the plant (bacteria harvested at 1 dpi of 7-, 14- and 21-day-old pea plants (Table 1; Additional file 2)); (iii) level of bacterial inoculum (10^3 ^or 10^8 ^colony forming units (CFU; 7 dpi of 7-day-old peas); Table 1; Additional file 3).

Incubating bacteria in the pea rhizosphere for 7 dpi was chosen as the standard incubation because it gave the highest number of three-fold or more differentially regulated genes (7 dpi (764) > 3 dpi (682) > 1 dpi (638); Additional file 1). Seven-day-old plants were chosen because this gave the largest number of three-fold or more differentially regulated genes (7-day-old plants (635) > 21-day-old (441) > 14-day-old (171); Additional file 2). In addition, 138 genes were specifically up-regulated in 7-day-old pea plants (Additional file 2), including many genes of interest (for example, *rhi *genes pRL10169-171, *cinI *(RL3378) and *nod *genes pRL100180, pRL100183, pRL100186-188), which we assume are induced by young, fast growing roots but not by those of older plants. An inoculum of 10^8 ^CFU rhizobia was chosen because it resulted in more differentially expressed genes (Additional file 3) and RNA recovery was more reliable.

With the standard conditions established *R. leguminosarum *bv. *viciae *Rlv3841 was inoculated at 10^8 ^CFU into the rhizosphere of 7-day-old pea, alfalfa or sugar beet plants and bacteria harvested 7 dpi. The gene induction pattern was compared against glucose-grown laboratory cultures, leading to an indirect comparison of rhizospheres (Table 1; Additional file 4). By contrast, relative levels of gene induction were also directly compared from bacteria isolated from the rhizospheres of two different plants (that is, pea:alfalfa, pea:sugar beet and alfalfa:sugar beet; Table 1; Additional files 4 and 5). Thus, the results of two independent methods could be compared.

**Figure 1:**
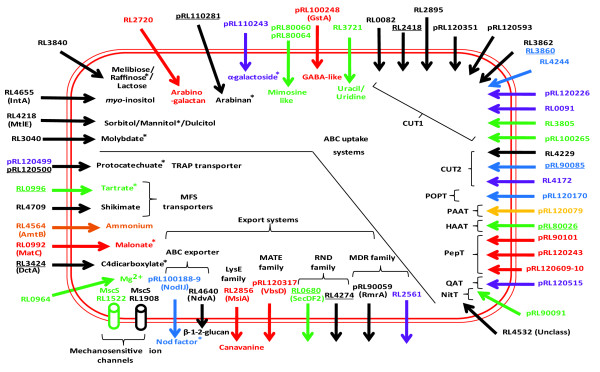
**Rhizosphere-induced genes in the Rlv3841 transportome**. Genes induced (by three-fold or more, *P *≤ 0.05) are color coded: black, all rhizospheres; green, pea-specific; red, alfalfa-specific; orange, sugar beet-specific; blue, legume-specific; purple, alfalfa and sugar beet; gold, pea and sugar beet. Genes are scored as elevated in more than one rhizosphere if they are up-regulated by three-fold or more in one and two-fold or more in one or two other rhizospheres (Additional file 8). Identified transported solutes are shown. Uncharacterized ABC uptake systems are classified according to Saier [12]. For uptake ABC transporter family: CUT1, carbohydrate uptake transporter 1; CUT2, carbohydrate uptake transporter 2; HAAT, hydrophobic amino acid transporter; MolT, molybdate transporter; NitT, nitrate/nitrite/cyanate transporter; PAAT, polar amino acid transporter; PepT, peptide/opine/nickel transporter; POPT, polyamine/opine/phosphonate transporter; QAT, quaternary amine transporter. Classification of ABC transporters is as follows: MolT, RL3040; CUT1, RL2418 (MtlE); CUT2, RL4655 (IntA), RL3840 and RL2720; PepT, pRL110281 and pRL110243; PAAT, pRL80060 and pRL80064; POPT, pRL100248; NitT, RL3721. Asterisks indicate a compound metabolized by an enzyme whose expression is elevated (Figure 2) or, in the case of Nod factor, synthesized for export (Figure 3). Abbreviations: ABC, ATP-binding cassette; MATE, multidrug and toxic compounds extrusion; MDR, multi-drug resistance; MFS, multi-facilitator superfamily; MscS, mechanosensitive channel small; RND, resistance-nodulation-cell division; TRAP, tripartite ATP-independent periplasmic. Genes underlined have been mutated and results of their competitiveness in the rhizosphere are shown in Additional file 8.

**Figure 2:**
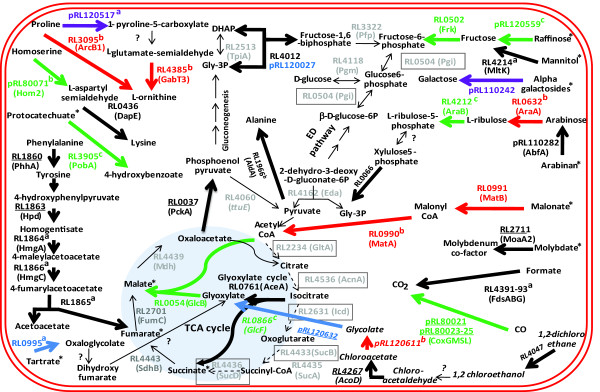
**Rhizosphere-induced genes in Rlv3841 metabolic pathways**. Bold lines show reactions encoded by genes induced (by three-fold or more, *P *≤ 0.05) and are color coded for the rhizospheres: black, all; green, pea-specific; red, alfalfa-specific; blue, legume-specific; purple, alfalfa and sugar beet. An induced gene is also considered to be elevated in further rhizospheres where expression was elevated by two-fold or more (Additional file 8). Where more than one enzyme carries out a reaction the color reflects the gene elevated in most rhizospheres. Asterisks indicate compounds imported by a transport system whose expression was elevated (Figure 1). ^a^Genes are part of an induced operon and elevated ≥ 1.3-fold; ^b^genes induced ≥ 3-fold, *P *≤ 0.11; ^c^genes elevated ≥ 2.2-fold, *P *≤ 0.05. Dotted lines and boxed genes show those down-regulated (≤ 0.3-fold, *P *≤ 0.05) relative to glucose-grown cells. Expression is considered to be down-regulated in all rhizospheres if ≤ 0.5-fold. Reactions carried out by genes slightly down-regulated (0.4- to 0.8-fold; Additional file 8) are shown by a thin grey line. The dichloroethane pathway shown in italics is speculative. Genes underlined have been mutated and results of their competitiveness in the rhizosphere are shown in Additional file 8. DHAP, dihydroxyacetone phosphate; ED, Entner-Doudoroff; TCA, tricarboxylic acid.

**Figure 3:**
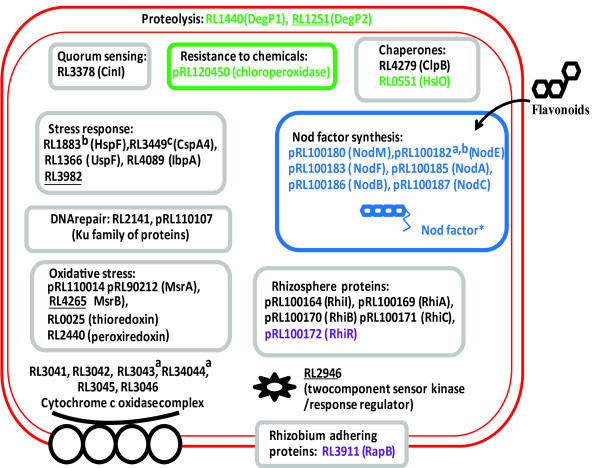
**Rhizosphere-induced genes for cellular functions in Rlv3841**. Genes elevated by three-fold or more (*P *≤ 0.05) are color coded for the rhizospheres: black, all; green, pea-specific; blue, legume-specific; purple, alfalfa and sugar beet; gold, pea and sugar beet. An induced gene is considered to be also elevated in further rhizospheres where expression was elevated by two-fold or more (Additional file 8). Asterisks indicate Nod factor is exported by a specific transport system whose expression was elevated (Figure 1). ^a^Genes are part of an induced operon and elevated ≥ 1.7-fold; ^b^genes induced ≥ 3-fold, *P *≤ 0.08; ^c^gene considered up-regulated in the pea rhizosphere (1.8-fold elevated). Genes underlined have been mutated and results of their competitiveness in the rhizosphere are shown in Additional file 8.

Increased gene expression was classified as general (that is, elevated in all plant rhizospheres) or specific, either for the rhizospheres of legumes or individual plant species (Additional files 4 and 6). Seventy of the 106 genes up-regulated in all rhizospheres tested compared to glucose-grown bacteria (Additional files 4 and 7) are annotated as hypothetical (compared to 27% of the genome); even permitting for a degree of mis-annotation, this suggests synthesis of proteins of novel function. A similar observation has been made for *Pseudomonas *[6]. As our purpose was to integrate information about metabolism and cellular function in the rhizosphere, we have avoided a tedious list of genes and instead distilled key features of bacterial life in the rhizosphere into diagrams for membrane transport (Figure 1), metabolism (Figure 2) and cellular activities (Figure 3) (data in Additional file 8).

In order to determine the importance of bacterial genes up-regulated in the rhizosphere, competition assays were performed in the pea rhizosphere between wild-type Rlv3841 and 46 strains, each mutated in one of these up-regulated genes (Additional file 8). These genes were chosen after the initial screen of genes up-regulated in the pea rhizosphere versus glucose grown laboratory cultures. Pea was used because it is the host plant for *R. leguminosarum*. However, two mutants were also tested in both pea and alfalfa rhizospheres because subsequent gene expression analysis showed they are specifically up-regulated in the pea rhizosphere. In these mutants it would be expected that any impairment in competition would be restricted to the pea rhizosphere. Mutants were scored with a rhizosphere colonization index (RCI); as described in Materials and methods, a RCI of 1 indicates equal competitiveness with Rlv3841, and the lower the RCI (down to 0.35), the less able the strain is to compete with Rlv3841 (Additional file 8). Thus, a low RCI indicates that the mutation is in a gene that is important for the strain to colonize the rhizosphere.

### General adaptation to the rhizosphere: cellular factors

Genes induced in all three plant rhizospheres reflect general life in the rhizosphere and we consider these before examining responses specific to one plant (Additional file 7). They include elevated expression of *rhiABC *(pRL100169-171) and *rhiI *(pRL100164), previously described as rhizosphere-induced genes [7], and the gene for autoinducer synthesis protein CinI (RL3378), which is involved in coordinating quorum-sensing regulation and biofilm formation (Figure 3). Quorum sensing is likely to be important in rhizosphere biology, where bacterial attachment is a key step in root colonization [7]. Expression of genes encoding an alternative *aa_3_*-type cytochrome *c *oxidase complex (RL3041-45) and a possibly associated cytochrome *c *(RL3046) were induced in the rhizosphere (Figure 3). This rhizosphere-induced cytochrome pathway, which is distinct from both the normal cytochrome *aa_3 _*complex found in laboratory cultured bacteria and the high affinity cytochrome *cbb_3 _*complex found in the N_2_-fixing nodule form of rhizobia [8], suggests a distinct redox environment in the rhizosphere. It may be that in the rhizosphere the level of available oxygen is lower than in shaken laboratory culture but higher than in the microaerophilic conditions found inside legume nodules.

### General adaptation to the rhizosphere: metabolism and transport

Up-regulation of genes encoding C4-dicarboxylate transport protein, DctA (RL3424; Figure 1), and PEP carboxykinase, PckA (RL0037; Figure 2), reveals increased organic acid metabolism in the rhizosphere. Induction of *pckA *is required for gluconeogenesis and indicates sugar synthesis. *R. leguminosarum *represses *pckA *when grown on organic acids with added sugar [9] so while sugars are present in the rhizosphere (that is, based on induction of sugar transporters), central metabolism is almost certainly dominated by catabolism of organic acids. Soils are rich in organic acids and they are the main carbon sources in the tomato rhizosphere [10]. Mutations in both *dctA *(RL3424) and *pckA *(RL0037) decreased the ability of *R. leguminosarum *to compete in the pea rhizosphere as shown by RCIs of 0.65 and 0.57, respectively (Additional file 8).

The glyoxylate cycle was induced showing that short chain (C2) organic acids are catabolized (Figure 2). C1 metabolism is important based on the induction of NAD^+^-dependent formate dehydrogenase (RL4391-3) in all rhizospheres (Figure 2). Formate induced this operon in a laboratory culture of Rlv3841 (Additional file 9) and is a carbon source for autotrophic growth of *S. meliloti *[11]. Formate dehydrogenase (RL4391-3) requires a Mo cofactor and the gene encoding MoaA2 (RL2711), involved in molybdenum cofactor biosynthesis, showed elevated expression (Figure 2). Mutation of *moaA2 *(RL2711) resulted in a RCI of 0.73 in the pea rhizosphere (Additional file 8). In addition, in all the rhizospheres tested there was induction of an ABC transporter solute binding protein (SBP; RL3040) from the MolT (molybdate transporter) family (ABC families are according to Saier [12]), which is likely to be part of an uptake system for molybdate (Figure 1; Additional file 10).

Aromatic compounds are important precursors or breakdown products of many plant compounds and can be used as a source of carbon by rhizosphere bacteria. Their presence in the rhizosphere is illustrated by induction of genes encoding transport systems for uptake of shikimate and protocatechuate. Shikimate is taken up by a multi-facilitator super-family (MFS) transporter (RL4709). Protocatechuate is imported by a TRAP transporter (pRL120499-pRL120500; Figure 1), which was identified by high level induction of pRL120498-500 in microarrays of cells grown in the presence of protocatechuate (Additional files 2 and 5). In the pea rhizosphere, mutation of pRL120500 led to a RCI of 0.72 (Additional file 8). Catabolism of aromatic compounds has also been shown to be important for *Pseudomonas putida *in the rhizosphere of *Zea mays *[13].

One of the strongest general metabolic responses in the rhizosphere was induction of genes encoding proteins involved in catabolism of phenylalanine and tyrosine (RL1860-6; Figure 2). These genes were also induced in free-living cells grown on phenylalanine (Additional file 9). The presence of phenylalanine in the rhizosphere probably results from its important role as a precursor for lignin synthesis by roots. Mutation of two genes encoding enzymes on this phenylalanine breakdown pathway (RL1860 and RL1863; Figure 2) led to two of the largest reductions in pea rhizosphere competitiveness (RCIs of 0.42 and 0.45, respectively; Additional file 8).

Common to all rhizospheres was induction of genes for uptake systems for inositol (IntA, RL4655) [8,14] and sorbitol/mannitol/dulcitol (MtlE, RL4218). Also elevated were genes encoding components of two previously uncharacterized systems. The first, RL3840, encodes a CUT1 (carbohydrate uptake transporter 1) family SBP likely to transport raffinose, melibiose and lactose based on 91% identity to SMb20931 from *S. meliloti*, whose expression was induced by these sugars [5]. The second, pRL110281, which encodes a PepT (peptide/opine/nickel transporter) family SBP, is clearly important in the pea rhizosphere since mutation of the gene led to a RCI of 0.44 (Additional file 8). The contiguous gene, pRL110282, encodes a product with putative α-N-arabinofuranosidase activity that could be responsible for removing arabinose subunits from arabinan. Based on this proximity, pRL110281 may import the arabinose polymer arabinan, or an oligosaccharide derived from it. Indeed, pRL110281 is unlikely to transport arabinose as its gene was not induced in laboratory cultures grown on arabinose (Additional file 9). Growth on arabinose did cause induction of genes encoding components of CUT2-family transporters, RL3615-6 and RL2377-8 (Additional files 9 and 10), neither of which was elevated in the rhizospheres tested.

Co-induction of transport systems and metabolic pathways provides additional evidence of the presence of a compound in the rhizosphere. The gene encoding mannitol dehydrogenase (MalK, RL4214), which converts mannitol to fructose, was elevated (Figure 2) along with those of a mannitol uptake system (MtlE, RL4218; Figure 1). Although *intA *(RL4655), encoding the *myo*-inositol transporter, was induced, genes for inositol catabolism were not. Induction of uptake genes may occur at lower substrate concentrations than for catabolic genes, and there are many examples in our data where catabolic genes were less induced than corresponding transport genes.

The genes encoding a CUT1 family ABC system (RL3860-2) were induced in all rhizospheres and although the solute specificity of this system is unknown, it clearly has a role in the pea rhizosphere as a mutation in RL3860 led to a RCI of 0.58 (Additional file 8). The transporter genes are surrounded by genes encoding predicted mandelate racemase/muconate lactonising proteins (RL3858, RL3864-66), a family of enzymes involved in breakdown of lignin-derived aromatic compounds, protocatechuate and catechol to intermediates of the citric acid cycle via the β-ketoadipate pathway. Although the transport genes were induced in all rhizospheres, of the genes encoding lignin breakdown enzymes only RL3864 was slightly elevated in the alfalfa rhizosphere (1.5-fold, *P *≤ 0.05) and RL3866 in the pea rhizosphere (1.4-fold, *P *≤ 0.05).

Examination of ABC transporters with unknown solute-specificity shows that 11 genes encoding CUT1 transporters were induced in plant rhizospheres (Figure 1). An increase of expression of CUT1 systems, which usually import oligosaccharides and their derivatives, is consistent with the presence in the rhizosphere of many different poly- and oligosaccharides. In addition, some members of the PepT class also transport oligosaccharides - for example, the α-galactoside (Agp) transporter (pRL110243; Figure 1) [15]. Genes for five PepT transporters were induced in these rhizospheres, with three of unknown solute-specificity induced only in the alfalfa rhizosphere (pRL90101, pRL120243 and pRL120609-10; Figure 1). This work supports the hypothesis that the large increase in the number of high-affinity ABC systems in rhizobia (and other α-proteobacteria) results from selective adaptation to the oligotrophic nature of soil and the rhizosphere.

### General adaptation to the rhizosphere: dealing with adversity

Plants produce antimicrobial agents (for example, phytoalexins) that bacteria must degrade or export. Plant-made antimicrobials such as halogenated hydrocarbons (for example, dichloroethane) could be dealt with by induction of RL4047 and RL4267, whose products may catalyze conversion of dichloroethane via chloroacetic acid to glycolate, with further degradation by the glyoxylate cycle (Figure 2). RL4267 shows 84% identity to a *Xanthobacter autotropicus *enzyme involved in 1,2-dichloroethane degradation [16]. Mutation of RL4267 resulted in a strain with reduced competitiveness in the pea rhizosphere (RCI = 0.47; Additional file 8). There have been descriptions of other haloalkanoate dehalogenases in *Rhizobium *sp. [17], suggesting halogenated hydrocarbons may act as antimicrobials around roots.

In addition to metabolic detoxification, expression of the multi-drug resistance (MDR) family efflux pump encoded by *rmrA *(pRL90059) was elevated (Figure 1). RmrA is a membrane fusion protein whose role is typically to dock an inner membrane exporter to a TolC-like protein that spans the periplasm and outer membrane. An *R. etli rmrA *mutant produced 40% less nodules on bean roots and had increased sensitivity to phytoalexins, flavonoids and salicylic acid [18]. Another membrane fusion protein elevated in all rhizospheres is encoded by RL4274, a RND (resistance-nodulation-cell division) multi-drug exporter (Figure 1). The importance of this system in the pea rhizosphere is demonstrated by a mutant of RL4274 having a RCI of 0.57 (Additional file 8).

Maintaining the correct osmotic environment is important for bacteria in any situation. In all rhizospheres there was elevated expression of *ndvA *(RL4640). NdvA is responsible for export of cyclic β-1-2-glucan to the bacterial periplasm and important in rhizobia for hypoosmotic regulation [19] (Figure 1). Expression of RL1908, encoding a small-conductance mechanosensitive ion channel (MscS), was also elevated in the rhizospheres examined (Figure 1) and is important in osmotic homeostasis. This suggests that the test microcosm was mildly hypoosmotic.

Elevation of expression of genes involved in response to stress occurred in all rhizospheres (Figure 3). Mutation of RL3982 and RL4265 (*msrB*), which encode general- and oxidative-stress proteins, reduced pea rhizosphere competitiveness (RCIs of 0.52 and 0.55, respectively; Additional file 8). Some of the largest effects on ability to compete in rhizospheres were shown by mutation of genes encoding proteins of unknown function; the photo reaction centre (PRC) family protein RL0913 and flavoprotein RL3366 had RCIs of 0.42 and 0.43, respectively, in the pea rhizosphere (Additional file 8). Mutation of RL2946, encoding part of a two-component sensor regulator (Figure 3), led to a RCI of 0.59 in the pea rhizosphere (Additional file 8).

### Specific adaptation to legume rhizospheres

The largest class of genes induced only in legume rhizospheres were the *nod *genes (*nodABCEFIJLMNO*), required for synthesis and export of nodulation factors (Figure 3). This acts as an exquisite internal control since nodulation factors are specifically induced in response to secretion of flavonoids by legumes [3]. There is also a legume rhizosphere-specific transporter encoded by pRL90085 (Figure 1) shown to be important in the pea rhizosphere as mutation led to a RCI of 0.52 (Additional file 8). Although the solute is unknown, it is probably a monosaccharide, as pRL90085 is in the CUT2 family.

### Specific adaptation to the pea rhizosphere

Increased expression of genes encoding enzymes of the glyoxylate cycle (RL0054, RL0866) only occurred in the pea rhizosphere. RL0054 (malate synthase) forms malate from glyoxylate and acetyl CoA while GlcF (RL0866) probably converts glycolate to glyoxylate (Figure 2). Thus, while C2 metabolism is elevated in all rhizospheres, it is particularly important in that of pea. Curiously, although the gene for isocitrate lyase (RL0761, *aceA*) was up-regulated in both alfalfa and sugar beet rhizospheres, indicating elevated C2 metabolism, expression of RL0054, encoding malate synthase, was only elevated in that of pea (Figure 2).

The gene for MFS transporter of tartrate (RL0996) was induced by three-fold or more in the pea rhizosphere (Figure 1; Additional file 7) while that for tartrate dehydrogenase (RL0995), which converts tartrate to oxaloglycolate for metabolism by the glyoxylate cycle, was only induced by legumes [20] (Figure 2). Mutation of RL0996, encoding the tartrate transporter, led to the largest effect on ability to compete in the pea rhizosphere (RCI = 0.35; Additional file 8). RL0996 was also induced 1.5-fold in the alfalfa rhizosphere, so although this falls below our two-fold cutoff, it suggests tartrate utilization may be important in legume rhizospheres (Additional file 8). However, tartrate may be more generally important as in *Agrobacterium vitis *the ability to utilize tartrate offered a selective advantage for growth on grapevine [21].

### The importance of pRL8 in the pea rhizosphere

**Figure 4:**
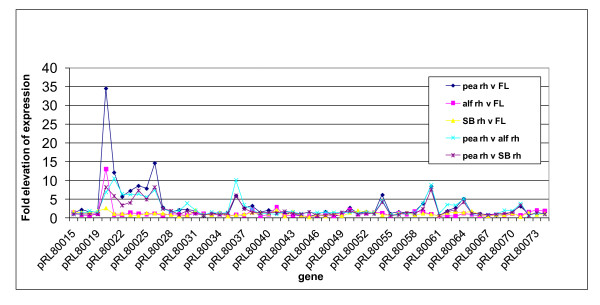
**Expression pattern of a pea rhizosphere specific region of pRL8**. Abbreviations: Pea rh, bacteria grown in the pea rhizosphere; FL, free-living bacteria; alf rh, bacteria grown in the alfalfa rhizosphere; SB rh, bacteria grown in the sugar beet rhizosphere.

*R. leguminosarum *Rlv3841 has a chromosome and six plasmids designated pRL7-pRL12, with pRL10 containing most nodulation and nitrogen fixation genes [22]. Although pea rhizosphere-induced genes from different parts of the genome have been discussed above, many genes on pRL8 are specifically up-regulated in the pea rhizosphere (Figure 4; Additional file 6). Indeed, 37% (11 genes) of the 30 genes elevated by three-fold or more specifically in the pea rhizosphere (using both direct and indirect comparisons (Additional file 7)) are encoded on pRL8. With a threshold of up-regulation of two-fold or more (*P *≤ 0.05), then 21 genes on pRL8 are pea rhizosphere-specific (15% of all genes on pRL8). By comparison, only three and two genes on pRL8 were up-regulated in alfalfa and sugar beet rhizospheres, respectively, and two genes were up-regulated in the legume rhizosphere. Since plasmid pRL8 is conjugative [22], it can easily transfer between rhizobia. Consistent with its heavy bias to genes important in the pea rhizosphere, pRL8 shows little colinearity (< 5%) with other sequenced rhizobial genomes [23]. BLAST analysis shows that of its 142 genes, 25% are found only in *R. leguminosarum *bv *viciae *and a further 42% are specific to rhizobia or related α-proteobacteria.

Genes on pRL8 that are pea rhizosphere-specific include a molybdenum-containing xanthine dehydrogenase-like carbon monoxide dehydrogenase, CoxMSL (pRL80023-25), together with accessory protein CoxG (pRL80021). Nearby are genes for proteins that may be involved in maturation of this complex: proteins involved in molybdopterin biosynthesis (pRL80034 and pRL80033 encode MoaA and MoeA-like proteins, respectively) and CoxI (pRL80038), which is needed for insertion of a molydopterin cofactor into a xanthine dehydrogenase. However, while CoxMSL (pRL80023-25) may be able to catalyze CO conversion to CO_2 _(Figure 2), in phylogenetic clustering these proteins form a separate clade from the biochemically characterized CO dehydrogenases, including one from *Bradyrhizobium japonicum *[24]. Mutation of pRL80021 (*coxG*) and pRL80023 (*coxM*) resulted in reduced competitiveness in the pea rhizosphere (RCIs of 0.44 and 0.73, respectively; Additional file 8). Since pRL80021 is up-regulated only in the pea rhizosphere, its mutation did not result in reduced competitiveness in the alfalfa rhizosphere (RCI = 0.97; Additional file 8).

Homoserine is abundant in pea root mucilage and can be utilized as a carbon source by *R. leguminosarum *[25]. Although the genes involved in catabolism of homoserine are uncharacterized in Rlv3841, pRL80071, which encodes a putative homoserine dehydrogenase (which catalyses conversion of homoserine to L-aspartate-semialdehyde), was specifically up-regulated in the pea rhizosphere (Figure 2).

Tryptophan is probably available in the rhizosphere [26]. The gene encoding N-formylkynurenine formidase (pRL80036) was six- to ten-fold elevated in the pea rhizosphere (Figure 4). It catalyzes release of formate and kynurenine from N-formylkynurenine, formed after the first step in tryptophan catabolism. The formate produced might be further metabolized to CO_2 _by a NAD^+^-containing short-chain dehydrogenase encoded by pRL80037, whose expression is 2.5- to 3.5-fold elevated (Figure 4).

Mimosine (β-3-hydroxy-4 pyridone amino acid), a toxic amino acid related to tyrosine, is produced by the tree-legume leucaena, which is nodulated by *Rhizobium *sp. TAL1145. *Rhizobium *sp. TAL1145 has a specific ABC importer for mimosine (MidABC) and an aminotransferase responsible for its degradation (MidD) [27]. An ABC importer (encoded by pRL80060/pRL80063-4; Figure 1), which shows 44 to 79% identity to MidABC [27], was induced in the pea rhizosphere. However, there is no protein in Rlv3841 with > 27% identity to the aminotransferase required for mimosine degradation. While the transporter encoded by pRL80060/pRL80063-4 is unlikely to transport mimosine, it may transport a similar amino acid. Expression of this system was also elevated in 7-day-old pea bacteroids [8]; thus, it may have a role in the symbiotic interaction between Rlv3841 and pea.

Also elevated specifically in pea rhizospheres were pRL80026-30, which encode proteins belonging to the HAAT (hydrophobic amino acid transporter) ABC family (Figure 1). Despite the fact that this transporter has been annotated as a LIV (leucine, isoleucine, valine) system, it could transport one or more aromatic amino acid(s) or homoserine. Mutation of pRL80026 resulted in a RCI of 0.69 in the pea rhizosphere (Additional file 8).

### Dealing with adversity in the pea rhizosphere

Export of plant toxins is likely to be important for successful growth in the rhizosphere. The gene encoding the RND family exporter RL4274 was induced 3.5- to 2.8-fold in the rhizospheres of alfalfa and sugar beet and 135-fold (*P *≤ 0.05) in pea (Figure 1). In addition, *secDF2 *(RL0680) encodes a membrane protein specifically induced to a very high level only in the pea rhizosphere (> 100-fold, *P *≤ 0.05; Figure 1). SecDF homologues belong to the RND exporter superfamily and RL0680 may participate in metabolite rather than protein export. It is clear that SecDF2 has a key role affecting competitiveness in the pea rhizosphere since a mutant in this gene had a RCI of 0.45 (Additional file 8). In contrast, in the alfalfa rhizosphere the same mutant had a RCI of 0.97 (Additional file 8), showing that mutation of *secDF2 *has no significant effect on its ability to compete with Rlv3841in the alfalfa rhizosphere.

The mildly hypoosmotic nature of the general rhizosphere microcosm was revealed by the induction of genes encoding cyclic β-1-2-glucan exporter (NdvA, RL4640) and a mechanosensitive ion channel (MscS, RL1908) (Figure 1). In addition, the gene for a second MscS (RL1522) was specifically induced in the pea rhizosphere (Figure 1), presumably fine-tuning the osmotic response.

### Specific adaptation to the alfalfa rhizosphere

A dicarboxylate may be a key carbon source in the alfalfa rhizosphere since genes for the malonate transporter (MatC, RL0992; Figure 1) and enzymes for malonate metabolism to acetyl CoA (MatA, RL0990, and MatB, RL0991; Figure 2) were induced. Alternatively, the malonate present in the alfalfa rhizosphere may need to be detoxified as it can act as an inhibitor of succinate dehydrogenase.

Canavanine is a toxic amino acid analogue of arginine found in seeds and exudates of leguminous plants [28]. The canavanine exporter MsiA from *Mesorhizobium tianshanense *shows 97% identity with RL2856. In Rlv3841, expression of RL2856 was specifically elevated in the alfalfa rhizosphere (Figure 1). Canavanine comprises 0.6 to 1.6% of the dry weight of alfalfa seeds and restricts growth of bacteria [28]. MsiA (RL2856) was slightly induced in vetch bacteroids (3.2-fold, *P *= 0.07) [8], suggesting that vetch releases some canavanine. MsiA is important for attachment to root hairs and survival in rhizospheres of canavanine-producing legumes [29]. The ability to deal with toxic canavanine may enable selection of MsiA-producing bacteria by these leguminous plants.

RL2720 encodes a CUT2 family SBP specifically induced in the alfalfa rhizosphere (Figure 1). From the up-regulation of expression of RL2720-2 (4- to 25-fold) in microarrays of cells grown in the presence of arabinogalactan (Additional file 9), this cluster of genes may encode an arabinogalactan transporter (Additional file 10). In addition, this gene cluster encodes two transketolases (RL2718-19). Expression of RL2719 was elevated (12-fold) in the alfalfa rhizosphere (Additional file 7), as was that of a short chain dehydrogenase (RL2725). The proteins encoded by these genes may have a role in arabinogalactan metabolism. These genes were not elevated in microarrays of cells grown on galactose or arabinose (Additional file 9), indicating a response only to the polysaccharide or to oligosaccharide break-down products (and not the monosaccharide constituents) of arabinogalactan.

### Specific adaptation to the sugar beet rhizosphere

Comparative analysis reveals that the sugar beet rhizosphere is N-limited. Expression of genes encoding glutamine synthetase II (GlnII, RL3549), the NH_4_^+ ^transporter AmtB (RL4564) (Figure 1) and PAAT (polar amino acid transporter) family importer pRL120079 (Figure 1) was elevated, as they were in all N-limited microarrays (Additional files 7 and 9). As these experiments were deliberately conducted in N-free plant growth medium, it is not surprising that the sugar beet rhizosphere was N-limited. However, legume rhizospheres were not N-limited, indicating release of nitrogen into the rhizosphere, possibly specifically because rhizobia were present.

### Response to root secretions compared with growth in the rhizosphere

As useful information about how micro-organisms respond to the rhizosphere can be obtained by incubating bacteria in root secretions [6], bacterial responses to constituent components were measured (Table 1). Pea root exudate was added to liquid-grown cells and microarray analysis showed 21 genes elevated by ≥ 3-fold (*P *≤ 0.05), 18 of which were also elevated by growth in medium containing the flavonoid hesperetin (27 genes elevated ≥ 3-fold, *P *≤ 0.05) (Additional file 6). Common induced genes, in addition to the *nod *and *rhi *gene clusters on pRL10 (*nodABCEFIJLMNO *and *rhiIABC*), include RL2418, encoding a CUT1 ABC transporter (and also induced three-fold or more in all rhizospheres and in the presence of acetoacetate, hydroxybenzoate and protocatechuate) (Additional files 6 and 9). Expression of RL2418 is clearly important for pea rhizosphere growth as a mutant showed much reduced competitiveness with Rlv3841 in the pea rhizosphere (RCI 0.43; Additional file 8).

Genes elevated under these two conditions (root exudate and hesperetin) form only a small proportion of those elevated in plant rhizospheres. One reason is that root secretions are very dilute and only induce genes responsive to low concentrations of bioactive compounds (for example, *nod *genes). Furthermore, growth in the rhizosphere involves a far more complex series of interactions, including attachment to roots, biofilm formation, contact with a complex array of plant macromolecules and cell-cell competition.

## Conclusions

Overall, the comparative transcriptome approach used here has revealed bacterial responses common to different plant rhizospheres, as well as mapping key general responses such as organic acid, C1-C2 and aromatic amino acid metabolism. In addition, it has highlighted specific bacterial adaptations to individual plants species and enabled identification of specific detoxification systems, such as that for canavanine in the alfalfa rhizosphere. Mutation of two genes (RL0680 and pRL80021) specifically induced in the pea rhizosphere only reduced competitiveness in the pea and not the alfalfa rhizosphere. A dramatic observation is the large number of genes located on plasmid pRL8 that are specifically induced in the pea rhizosphere. This is particularly exciting and suggests there may be a wealth of plant rhizosphere-specific plasmids or chromosomal islands to be revealed by emerging high-throughput sequencing projects.

## Materials and methods

### Bacterial and plant growth

*R. leguminosarum *strains were grown either in tryptone yeast [30] or acid minimal salts as described [31]. Strains, plasmids and oligonucleotide primers are described in Additional file 11. Seeds of pea (*Pisum sativa *cv Avola), alfalfa (*Medicago sativa*) or sugar beet (*Beta vulgaris*) were surface sterilized and grown in 50 ml Falcon tubes containing autoclaved washed vermiculite and N-free rooting solution [32]. Growth was at 23°C with a 16-h/8-h light/dark cycle for 7, 14 or 21 days before inoculation with Rlv3841 (10^3 ^or 10^8 ^CFU). After 1, 3 and 7 days shoots were removed and roots vortexed (5 minutes) in 6 ml sterile water and 12 ml RNA Protect (Qiagen, Crawley, West Sussex, UK). Insoluble material was removed by filtration through four layers of sterile muslin and centrifugation (160 × *g*, 1 minute, 4°C). Bacteria were recovered by centrifugation (12,000 × *g*, 10 minutes, 4°C) and re-suspended (200 μL 10 mM Tris-HCl, pH 8).

### RNA isolation and microarray analysis

Total RNA was extracted, quantified, amplified and hybridized to microarrays as described previously [8]. Results were analyzed using GeneSpring 7.2. (Agilent Technologies, Santa Clara, CA, USA) as described previously [8]. In brief, labeling, hybridization and scanning were as previously described, spot recognition was performed with Bluefuse (BlueGnome Limited, Cambridge, UK) and data were imported into GeneSpring 7.2 (Silicon Genetics, Redwood, CA, USA). The local background value was subtracted from the intensity of each spot and a Lowess normalization applied to the slide. Log ratios of expression and *P*-values were determined in GeneSpring 7.2. To establish a standard technique for sampling (that is, pea plant age on inoculation, harvest time dpi, inoculum size) preliminary experiments were performed (Table 1). Pea plant age was varied over 7, 14 and 21 days with bacterial harvest at 1 dpi (Additional file 1). Following inoculation of 7-day-old peas, the bacterial harvest was assessed at 1, 3 and 7 dpi (Additional file 2). The effect of Rlv3841 inoculum size (10^3 ^versus 10^8 ^CFU) was analyzed at 7 dpi of 7-day-old peas (E-MEXP-2854) (Table 1; Additional file 3). Standard conditions established were inoculation of 7-day-old plants with 10^8 ^CFU followed by bacterial harvest at 7 dpi.

Using the standard assay conditions, the first method used to compare rhizospheres was microarray analysis of bacteria grown in a rhizosphere (that of pea, alfalfa or sugar beet) against glucose-grown laboratory cultures (leading to an indirect comparison between rhizospheres). Five independent biological replicates were used for pea and three for alfalfa and sugar beet (Table 1). A second method of analysis was comparison of samples isolated from two different rhizospheres (direct comparison) each with four independent biological replicates taken at 7 dpi of 7-day-old plants (Table 1; Additional files 4 and 5).

Gene expression for selected genes was confirmed by quantitative RT-PCR performed in triplicate using the QuantiTect SYBR Green PCR Kit (Qiagen) on an MJ Mini cycler MiniOpticon Real-Time PCR Detection System (Bio-Rad, Hemel Hempstead, Hertfordshire, UK) as previously described [33]. Primers are given in Additional file 11. The data were analyzed by the relative quantification method (comparative C_T _method (ΔΔC_T_)) to calculate the fold expression [34,35]. Expression levels were normalized against *mdh *and analyzed with REST [34]. The results from the two normalization procedures were not significantly different (Additional file 12).

### Isolation of integration mutants and competition studies

*R. leguminosarum *bv *viciae *300 (Rlv300, Str^s^) is the parent of streptomycin-resistant (Str^r^) derivative Rlv3841. Mutations were made in 46 up-regulated genes in Rlv300 using plasmid integration (leading to neomycin-resistant (Neo^r^) mutant colonies) as described [8] (Additional file 8). To assess bacterial competiveness, 7-day-old pea or alfalfa seedlings (*n *= 8) were inoculated with a Neo^r ^mutant (10^4 ^CFU) and wild-type Str^r ^Rlv3841 (10^3 ^CFU). The 10:1 ratio of mutant to wild type was used as the wild type increases its frequency very effectively against strains with attenuated competitiveness [33], enabling detection of subtle decreases in competitiveness. After 7 days growth, recovered bacteria were serially diluted and CFU scored on tryptone yeast agar with trimethoprim (10 μg ml^-1^) and nystatin (50 μg ml^-1^) with either Str (500 μg ml^-1^; selects Rlv3841) or Neo (80 μg ml^-1^; selects mutant in Rlv300 background) as described [33]. A *nifH *mutant was used as a negative control as it is only expressed in nodules and is unaffected in its ability to compete in the rhizosphere, while a *thiM *mutant (thiamine auxotroph) was used as a positive control because it is severely attenuated for competitiveness in the pea rhizosphere [33]. To establish a baseline competition between wild-type strains, where no selective advantage is expected, Rlv300 was inoculated at ten-fold excess over Rlv3841. Rlv3841 was recovered at 9.7 ± 0.28% (mean ± standard error of the mean, *n *= 51; Additional file 8), close to the theoretical 9.1%. Similarly, for the positive (*thiM*) and negative (*nifH*) controls, Rlv3841 was recovered from the rhizosphere at 71.6 ± 1.6% and 10.0 ± 0.9% (mean ± standard error of the mean), respectively. These data have been expressed as RCI = 1/[(Percentage Rlv3841 recovered versus mutant)/(Percentage Rlv3841 recovered versus Rlv300)]. The RCI for the *thiM *mutant was 0.14 (1/(71.7%/9.7%)) and for *nifH *was 0.97 (1/(10.0%/9.7%)). RCIs for all mutants are shown in Additional file 8 and range from 0.35 to 1.1. As the RCIs for even the most affected mutants were not as low as that for the thiamine auxotroph (*thiM*), it was concluded that although no mutant strain was crippled for growth in the rhizosphere, several were significantly reduced in ability to colonize the rhizosphere in competition with wild-type bacteria.

## Abbreviations

ABC: ATP-binding cassette; CFU: colony forming unit; CUT: carbohydrate uptake transporter; dpi: days post-inoculation; MFS: multi-facilitator superfamily; MolT: molybdate transporter; MscS: mechanosensitive channel small; PepT: peptide/opine/nickel transporter; RCI: relative colonization index; RND: resistance-nodulation-cell division; SBP: solute binding protein; TRAP: tripartite ATP-independent periplasmic.

## Authors' contributions

VKR participated in the design of the study, the microarray studies, the data analysis and drafted the manuscript. AKE participated in the design of the study, the data analysis and drafted the manuscript. RK participated in the microarray studies. JAD participated in the design of the study and drafted the manuscript. PSP participated in the design of the study, the data analysis and drafted the manuscript. All authors read and approved the final manuscript.

## Supplementary Material

Additional file 1 **Figure S2 - effect of length of incubation in the pea rhizosphere. (a, b) **Venn diagrams of Rlv3841 genes up-regulated (a) and down-regulated (b) in the pea rhizosphere at 1, 3 and 7 dpi of 7-day-old plants. Total genes differentially regulated in each rhizosphere are shown in brackets. Venn diagrams were drawn in GeneSpring by selecting differentially regulated genes (by three-fold or more, filtered on confidence *P *≤ 0.05) for each condition. Click here for file

Additional file 2 **Figure S3 - effect of plant age. (a, b) **Venn diagrams of Rlv3841 genes up-regulated (a) and down-regulated (b) in the rhizosphere at 1 dpi of 7-, 14- and 21-day-old pea plants. Total genes differentially regulated in each rhizosphere are shown in brackets. Venn diagrams were drawn in GeneSpring following selection of differentially regulated genes (by three-fold or more, filtered on confidence *P *≤ 0.05) for each condition. Click here for file

Additional file 3 **Figure S4 - effect of size of inoculation of the pea rhizosphere. (a, b) **Venn diagrams of Rlv3841 genes up-regulated (a) and down-regulated (b) in the pea rhizosphere at 1 dpi of 7-day-old plants with 10^3 ^and 10^8 ^CFU. Total genes differentially regulated in each rhizosphere are shown in brackets. Venn diagrams were drawn in GeneSpring by selecting differentially regulated genes by three-fold or more, filtered on confidence *P *≤ 0.05) for each condition. Click here for file

Additional file 4 **Figure S1 - Venn diagrams of differentially regulated Rlv3841 genes (by three-fold or more, *P *≤ 0.05)**. **(a) **An indirect comparison of rhizospheres in which pea, alfalfa and sugar beet rhizospheres are investigated using comparison with glucose-grown free-living cells (i) up-regulated and (ii) down-regulated. **(b) **Up-regulated in direct comparison with (i) pea, alfalfa and sugar beet rhizospheres and (ii) legume and sugar beet rhizospheres. Total genes differentially regulated in each rhizosphere are shown in brackets and listed for (a) in Additional file 7. Of the 106 genes up-regulated by all three plant rhizospheres (a(i); Additional file 7), 66% are annotated as hypothetical and 15% are membrane proteins or concerned with cell surface. The 184 genes down-regulated in all rhizospheres (a(ii); Additional file 7) include those for proteins involved in general cellular functions of bacterial motility and chemotaxis (6%), tRNA and DNA synthesis, chromosome and plasmid replication, cell division, protein export by the Sec system, electron transport and formation of ATP. In addition, 12% are annotated as hypothetical, 13% are membrane proteins or concerned with cell surface and 16% are ribosomal proteins. Components of two ABC transport systems that import glucose (RL3624-5, RL4252) [8] are down-regulated in all rhizospheres in comparison with glucose-grown cells (Additional file 7). From direct comparison of the pea rhizosphere with those of alfalfa and sugar beet (b(i)) there are 30 pea rhizosphere-specific genes (listed in Additional file 7). There are 9 legume rhizosphere-specific genes up-regulated in both the pea and alfalfa rhizosphere compared to that of sugar beet (b(ii)). Abbreviations: SB, sugar beet. Click here for file

Additional file 5 **Figure S5 - experimental design for direct comparison of Rlv3841 grown in three different rhizospheres**. **(a) **A single set for the direct comparison experiment; two biological replicates from each rhizosphere sample were extracted and amplified. From each amplified RNA sample, an equal amount (15 μg) of amplified RNA was taken and labeled with Cy3 and Cy5 separately. Equal amounts of labeled cDNAs were used for each microarray experiment. A second set was performed before analysis, yielding four biological replicates per rhizosphere. **(b) **Design for direct two-color experiments. **(c) **A table summarizing rhizosphere microarray experiments. Click here for file

Additional file 6 **Table S1 - genes whose expression was differentially regulated by three-fold or more compared to free-living Rlv3841 isolated from various conditions**. The conditions from which Rlv3841 were isolated were: the presence of hesperetin or pea exudate; the rhizospheres of 7-, 14- and 21-day-old pea plants at 1 dpi; the rhizosphere of 7-day-old pea plants at 1 and 3 dpi; and the rhizosphere of 7-day-old pea plants at 7 dpi inoculated with 10^3 ^CFU. The table also lists genes whose expression was differentially regulated by three-fold or more in the rhizosphere at 7 dpi of 7-day-old plants of pea versus alfalfa, pea versus sugar beet and alfalfa versus sugar beet. Click here for file

Additional file 7 **Table S2 - genes whose expression was differentially regulated by three-fold or more in the rhizospheres of pea, alfalfa and sugar beet at 7 dpi of 7-day-old plants compared to free-living Rlv3841**. In addition, genes specifically up-regulated in the pea rhizosphere are shown compared to free-living cells and those in the alfalfa rhizosphere and the sugar beet rhizosphere. Click here for file

Additional file 8 **Table S4 - data used to draw Figures **1, 2** and **3**and results for competition in pea and alfalfa rhizospheres of mutants compared with Rlv3841**. Click here for file

Additional file 9 **Table S3 - genes whose expression was differentially regulated by three-fold or more compared to free-living Rlv3841 when grown in the presence of formate, protocatechuate, 4-hydroxybenzoate, phenylalanine, proline, L-arabinose, galactose, arabinogalactan and under conditions of N-limitation**. Click here for file

Additional file 10 **Table S5 - identification of putative substrates for ABC, TRAP and MFS transporters of *R. leguminosarum *3841**. Click here for file

Additional file 11 **Table S6 - strains, plasmids and primers**. Click here for file

Additional file 12 **Table S7 - real time-quantitative reverse transcription PCR and log fold values calculated by the comparative C_T _method**. Click here for file
