# Supplementary material for: Adaptation of Rhizobium leguminosarum to pea, alfalfa and sugar beet rhizospheres investigated by comparative transcriptomics
Source: Genome Biol. 2011 Oct 21;12(10):R106. doi: 10.1186/gb-2011-12-10-r106 (PMC3333776; doi:10.1186/gb-2011-12-10-r106)
Supplement: Additional file 5 — Figure S5 - experimental design for direct comparison of Rlv3841 grown in three different rhizospheres. (a) A single set for the direct comparison experiment; two biological replicates from each rhizosphere sample were extracted and amplified. From each amplified RNA sample, an equal amount (15 μg) of amplified RNA was taken and labeled with Cy3 and Cy5 separately. Equal amounts of labeled cDNAs were used for each microarray experiment. A second set was performed before analysis, yielding four biological replicates per rhizosphere. (b) Design for direct two-color experiments. (c) A table summarizing rhizosphere microarray experiments. [file gb-2011-12-10-r106-S5.DOC]

7

8

9

10

11

12

**Repeat arrays**

1. **(C)**

| **Arrays** | **Cy3** | **Cy5** |
| --- | --- | --- |
| 1, 7 | 7d Alfalfa 7dpi | 7d Pea 7dpi |
| 2, 8 | 7d Sugar-beet 7dpi | 7d Pea 7dpi |
| 3, 9 | 7d Pea 7dpi | 7d Alfalfa 7dpi |
| 4, 10 | 7d Sugar-beet 7dpi | 7d Alfalfa 7dpi |
| 5, 11 | 7d Alfalfa 7dpi | 7d Sugar-beet 7dpi |
| 6, 12 | 7d Pea 7dpi | 7d Sugar-beet7dpi |

**Fig. S5. Experimental** **design for direct comparison of Rlv3841 grown in three different rhizospheres.** **(A)** For a direct comparison experiment, two biological replicates from each rhizosphere sample were extracted and amplified. From each amplified RNA sample, an equal amount (15μg) of amplified RNA was taken and labelled with Cy3 and Cy5 separately. Equal amounts of labelled cDNAs were used for each microarray experiment (arrays 1-6). A second set of biologically independent samples were taken and microarrays performed (arrays 7-12) before analysis, resulting in 4 biological replicates per rhizosphere comparison (e.g. for pea and alfalfa rhizospheres; arrays 1, 3, 7 and 9). **(B)** Design for direct two-colour experiments and **(C)** table summarising rhizosphere microarray experiments.
