# Supplementary material for: Adaptation of Rhizobium leguminosarum to pea, alfalfa and sugar beet rhizospheres investigated by comparative transcriptomics
Source: Genome Biol. 2011 Oct 21;12(10):R106. doi: 10.1186/gb-2011-12-10-r106 (PMC3333776; doi:10.1186/gb-2011-12-10-r106)
Supplement: Additional file 6 — Table S1 - genes whose expression was differentially regulated by three-fold or more compared to free-living Rlv3841 isolated from various conditions. The conditions from which Rlv3841 were isolated were: the presence of hesperetin or pea exudate; the rhizospheres of 7-, 14- and 21-day-old pea plants at 1 dpi; the rhizosphere of 7-day-old pea plants at 1 and 3 dpi; and the rhizosphere of 7-day-old pea plants at 7 dpi inoculated with 103 CFU. The table also lists genes whose expression was differentially regulated by three-fold or more in the rhizosphere at 7 dpi of 7-day-old plants of pea versus alfalfa, pea versus sugar beet and alfalfa versus sugar beet. [file gb-2011-12-10-r106-S6.DOC]

**Table S6. Strains, plasmids and primers**

| **Strain** | **Genotype** | **Reference** |
| --- | --- | --- |
| Rlv300 | *R. leguminosarum* bv. *viciae* wild type (Str^s^) | (Johnston and Beringer, 1975) |
| Rlv3841 | Str^r^ derivative of *R. leguminosarum* bv. *viciae* strain Rlv300 | (Johnston and Beringer, 1975) |
| RU4222 | Rlv300 *RL1911*::pk19; Neo^r^ | This work |
| RU4229 | Rlv300 *RL0913*::pk19; Neo^r^ | This work |
| RU4230 | Rlv300 *RL3130*::pk19; Neo^r^ | This work |
| RU4231 | Rlv300 *RL3366*::pk19; Neo^r^ | This work |
| RU4232 | Rlv300 *RL4267*::pk19; Neo^r^ | This work |
| RU4233 | Rlv300 *RL3016*:: pk19; Neo^r^ | This work |
| RU4234 | Rlv300 *pRL110199*::pk19; Neo^r^ | This work |
| RU4235 | Rlv300 *RL1694*::pk19; Neo^r^ | This work |
| RU4247 | Rlv300 *pRL120632*::pk19; Neo^r^ | This work |
| RU4248 | Rlv300 *pRL80026*::pk19; Neo^r^ | This work |
| RU4249 | Rlv300 *RL2711*::pk19; Neo^r^ | This work |
| RU4250 | Rlv300 *pRL80023*::pk19; Neo^r^ | This work |
| RU4251 | Rlv300 *RL2469*::pk19; Neo^r^ | This work |
| RU4252 | Rlv300 *RL4265*::pk19; Neo^r^ | This work |
| RU4253 | Rlv300 *pRL80054*::pk19; Neo^r^ | This work |
| RU4254 | Rlv300 *RL1251*::pk19; Neo^r^ | This work |
| RU4255 | Rlv300 *RL3272*::pk19; Neo^r^ | This work |
| RU4256 | Rlv300 *pRL90085*::pk19; Neo^r^ | This work |
| RU4257 | Rlv300 *RL2259*::pk19; Neo^r^ | This work |
| RU4258 | Rlv300 *RL3424*::pk19; Neo^r^ | This work |
| RU4259 | Rlv300 *RL1860*::pk19; Neo^r^ | This work |
| RU4260 | Rlv300 *RL4274*::pk19; Neo^r^ | This work |
| RU4261 | Rlv300 *RL3860*::pk19; Neo^r^ | This work |
| RU4262 | Rlv300 *RL1297*::pk19; Neo^r^ | This work |
| RU4263 | Rlv300 *RL0996*::pk19; Neo^r^ | This work |
| RU4265 | Rlv300 *RL2418*::pk19; Neo^r^ | This work |
| RU4266 | Rlv300 *pRL120479*::pk19; Neo^r^ | This work |
| RU4267 | Rlv300 *RL1485*::pk19; Neo^r^ | This work |
| RU4268 | Rlv300 *pRL110281*::pk19; Neo^r^ | This work |
| RU4269 | Rlv300 *RL1863*::pk19; Neo^r^ | This work |
| RU4270 | Rlv300 *RL0787*::pk19; Neo^r^ | This work |
| RU4271 | Rlv300 *pRL80021*::pk19; Neo^r^ | This work |
| RU4272 | Rlv300 *RL0540*::pk19; Neo^r^ | This work |
| RU4273 | Rlv300 *RL0274*::pk19; Neo^r^ | This work |
| RU4274 | Rlv300 *RL0037*::pk19; Neo^r^ | This work |
| RU4295 | Rlv300 *pRL120500*::pk19; Neo^r^ | This work |
| RU4296 | Rlv300 *pRL100268*::pk19; Neo^r^ | This work |
| RU4297 | Rlv300 *RL3982*::pk19; Neo^r^ | This work |
| RU4298 | Rlv300 *RL0680*::pk19; Neo^r^ | This work |
| RU4308 | Rlv300 *RL1172*::pk19; Neo^r^ | This work |
| RU4309 | Rlv300 *pRL90055*::pk19; Neo^r^ | This work |
| RU4310 | Rlv300 *RL2946*::pk19; Neo^r^ | This work |
| RU4311 | Rlv300 *pRL100444*::pk19; Neo^r^ | This work |
| RU4312 | Rlv300 *pRL120724*::pk19; Neo^r^ | This work |
| RU4317 | Rlv300 *pRL110423*::pk19; Neo^r^ | This work |
| RU4318 | Rlv300 *RL3186*::pk19; Neo^r^ | This work |
| RU4358 | Rlv300 *pRL110443*::pk19; Neo^r^ | This work |
| RU4360 | Rlv300 *pRL100162*::pk19; Neo^r^ | This work |
| *E. coli* Fusion-Blue | *endA1 hsdR17(r_k12_^-^, m_k12_^+^) supE44 thi-1gyrA96 relA1 lacF’[proA^+^ B^+^ lacI^q^Z*Δ*M15::Tn10(tet^R^ )]* | Clontech laboratories |
| **Plasmids** |  |  |
| pK19mob | pK19mob pUC19 derivative *lacZ mob*; Neo^r^ | (Schaf*er et a*l., 1994) |
| pRK2013 | ColEI replicon with RK2 tra genes; Km^r^ | (Figurski and Helinski, 1979) |
| pRU2021 | *RL0913* PCR amplified with p1214 and p1215, cloned in pK19mob and mapped with p1216 on integration into Rlv300 | This work |
| pRU2024 | *RL3130* PCR amplified with p1223 and p1224, cloned in pK19mob and mapped with p1225 on integration into Rlv300 | This work |
| pRU2025 | *RL3366* PCR amplified with p1226 and p1227, cloned in pK19mob and mapped with p1228 on integration into Rlv300 | This work |
| pRU2033 | *RL4267* PCR amplified with p1175 and p1176, cloned in pK19mob and mapped with p1177 on integration into Rlv300 | This work |
| pRU2034 | *RL3016* PCR amplified with p1071 and p1072, cloned in pK19mob and mapped with p1527 on integration into Rlv300 | This work |
| pRU2038 | *RL0680* PCR amplified with p1097 and p1098, cloned in pK19mob and mapped with p1529 on integration into Rlv300 | This work |
| pRU2042 | *pRL110199* PCR amplified with p1093 and p1094, cloned in pK19mob and mapped with p1526 on integration into Rlv300 | This work |
| pRU2044 | *RL1694* PCR amplified with p1101 and p1102, cloned in pK19mob and mapped with p1524 on integration into Rlv300 | This work |
| pRU2047 | *RL0037* PCR amplified with BD_pckA_for and BD_pckA_rev, cloned in pK19mob and mapped with p1370 on integration into Rlv300 | This work |
| pRU2053 | *pRL100444* PCR amplified with p1545 and p1546, cloned in pK19mob and mapped with p1547 on integration into Rlv300 | This work |
| pRU2056 | *pRL100162* PCR amplified with p1208 and p1209, cloned in pK19mob and mapped with p1210 on integration into Rlv300 | This work |
| pRU2082 | *RL1911* PCR amplified with BD_RL1911_for and BD_RL1911_rev, cloned in pK19mob and mapped with p1523 | This work |
| pRU2100 | *RL2711* PCR amplified with p1422 and p1423, cloned in pK19mob and mapped with p1424 on integration into Rlv300 | This work |
| pRU2101 | *pRL80023* PCR amplified with p1431 and p1432, cloned in pK19mob and mapped with p1433 on integration into Rlv300 | This work |
| pRU2102 | *RL1251* PCR amplified with p1392 and p1393, cloned in pK19mob and mapped with p1394 on integration into Rlv300 | This work |
| pRU2103 | *RL3272* PCR amplified with p1389 and p1390, cloned in pK19mob and mapped with p1391 on integration into Rlv300 | This work |
| pRU2105 | *pRL120632* PCR amplified with p1095 and p1096, cloned in pK19mob and mapped with p1525 on integration into Rlv300 | This work |
| pRU2107 | *pRL90085* PCR amplified with p1419 and p1420, cloned in pK19mob and mapped with p1421 on integration into Rlv300 | This work |
| pRU2108 | *RL2259* PCR amplified with p1440 and p1441, cloned in pK19mob and mapped with p1442 on integration into Rlv300 | This work |
| pRU2109 | *RL1485* PCR amplified with p1470 and p1471, cloned in pK19mob and mapped with p1472 on integration into Rlv300 | This work |
| pRU2110 | *pRL110281* PCR amplified with p1383 and p1384, cloned in pK19mob and mapped with p1385 on integration into Rlv300 | This work |
| pRU2111 | *RL3424* PCR amplified with p1401 and p1402, cloned in pK19mob and mapped with p1403 on integration into Rlv300 | This work |
| pRU2112 | *RL1863* PCR amplified with p1416 and p1417, cloned in pK19mob and mapped with p1418 on integration into Rlv300 | This work |
| pRU2113 | *RL1860* PCR amplified with p1437 and p1438, cloned in pK19mob and mapped with p1439 on integration into Rlv300 | This work |
| pRU2114 | *pRL80021* PCR amplified with p1461 and p1462, cloned in pK19mob and mapped with p1463 on integration into Rlv300 | This work |
| pRU2115 | *RL4274* PCR amplified with p1410 and p1411, cloned in pK19mob and mapped with p1412 on integration into Rlv300 | This work |
| pRU2116 | *pRL120500* PCR amplified with p1413 and p1414, cloned in pK19mob and mapped with p1415 on integration into Rlv300 | This work |
| pRU2117 | *RL3860* PCR amplified with p1428 and p1429, cloned in pK19mob and mapped with p1430 on integration into Rlv300 | This work |
| pRU2118 | *RL1297* PCR amplified with p1443 and p1444, cloned in pK19mob and mapped with p1445 on integration into Rlv300 | This work |
| pRU2119 | *pRL110268* PCR amplified with p1473 and p1474, cloned in pK19mob and mapped with p1475 on integration into Rlv300 | This work |
| pRU2120 | *RL0996* PCR amplified with p1395 and p1396, cloned in pK19mob and mapped with p1397 on integration into Rlv300 | This work |
| pRU2121 | *RL0540* PCR amplified with p1407 and p1408, cloned in pK19mob and mapped with p1409 on integration into Rlv300 | This work |
| pRU2123 | *RL0274* PCR amplified with p1455 and p1456, cloned in pK19mob and mapped with p1457 on integration into Rlv300 | This work |
| pRU2124 | *pRL90055* PCR amplified with p1464 and p1465, cloned in pK19mob and mapped with p1466 on integration into Rlv300 | This work |
| pRU2125 | *RL2418* PCR amplified with p1404 and p1405, cloned in pK19mob and mapped with p1406 on integration into Rlv300 | This work |
| pRU2126 | *pRL120479* PCR amplified with p1425 and p1426, cloned in pK19mob and mapped with p1427 on integration into Rlv300 | This work |
| pRU2127 | *RL3982* PCR amplified with p1449 and p1450, cloned in pK19mob and mapped with p1451 on integration into Rlv300 | This work |
| pRU2128 | *pRL80026* PCR amplified with p1398 and p1399, cloned in pK19mob and mapped with p1400 on integration into Rlv300 | This work |
| pRU2129 | *pRL110423* PCR amplified with p1446 and p1447, cloned in pK19mob and mapped with p1448 on integration into Rlv300 | This work |
| pRU2130 | *RL2469* PCR amplified with p1478 and p1479, cloned in pK19mob and mapped with p1480 on integration into Rlv300 | This work |
| pRU2131 | *RL4265* PCR amplified with p1458 and p1459, cloned in pK19 and mapped with p1460 on integration into Rlv300 | This work |
| pRU2132 | *pRL80054* PCR amplified with p1467 and p1468, cloned in pK19mob and mapped with p1469 on integration into Rlv300 | This work |
| pRU2133 | *RL0787* PCR amplified with p1452 and p1453, cloned in pK19mob and mapped with p1454 on integration into Rlv300 | This work |
| pRU2166 | *RL1172* PCR amplified with p1548 and p1549, cloned in pK19 and mapped with p1550 on integration into Rlv300 | This work |
| pRU2167 | *RL2946* PCR amplified with p1551 and p1552, cloned in pK19mob and mapped with p1553 on integration into Rlv300 | This work |
| pRU2168 | *pRL120724* PCR amplified with p1557 and p1558, cloned in pK19mob and mapped with p1559 on integration into Rlv300 | This work |
| pRU2170 | *RL3186* PCR amplified with p1560 and p1561, cloned in pK19mob and mapped with p1562 on integration into Rlv300 | This work |
| pRU2187 | *pRL110443* PCR amplified with p1636 and p1637, cloned in pK19mob and mapped with p1638 on integration into Rlv300 | This work |
|  |  |  |
| **Primers** |  |  |
| BD_pckA_for | TGATTACGCCAAGCTTCGCTGTTCATCCGCAATCT |  |
| BD_pckA_rev | GCAGGCATGCAAGCTGTTTTCAGTCAGCGACCCGT |  |
| BD_RL1911_for | TGATTACGCCAAGCTCCGGCGCGGATATCGGTCTGC |  |
| BD_RL1911_rev | GCAGGCATGCAAGCTATGAGATTGTAGCCGTCGAG |  |
| p1071 | TTTTAAGCTTAGGTGTGGCCATTGTCGC |  |
| p1072 | TTTTCTAGATAGTCGGGATGCTCGCCA |  |
| p1093 | TTTTAAGCTTAGCAGTTCAATCCCACGGC |  |
| p1094 | TTTTCTAGAATCAGCATCGCAGGCAGC |  |
| p1095 | TTTTAAGCTTGCTACCTGATCACCTGGA |  |
| p1096 | TTTTCTAGATTGATGTTGGCGATGACG |  |
| p1097 | TTTTAAGCTTAGCCAGAACCAGATCGTC |  |
| p1098 | TTTTCTAGAGGCCTTCAGCTTGGTCAC |  |
| p1101 | TTTTAAGCTTGCTCCTGGGGCGATATCA |  |
| p1102 | TTTTCTAGAGACAGCGGCGCAAGGTTG |  |
| p1208 | TGATTACGCCAAGCTTTGAAGTTCGGCAAGCAT |  |
| p1209 | GCAGGCATGCAAGCTAGTCCACTACATCCCAAA |  |
| p1210 | TGTCACCGCCGAAAACGATG |  |
| p1214 | TGATTACGCCAAGCTGTCGAGCCAGTTGCCGTG |  |
| p1215 | GCAGGCATGCAAGCTTGTTTGCCACGGCTTTCG |  |
| p1216 | GTCGGCTTCGATCCGCATCG |  |
| p1223 | TGATTACGCCAAGCTGTAGCCGCCGGCTTCGTG |  |
| p1224 | GCAGGCATGCAAGCTAAGACCGCGCCCGACGAT |  |
| p1225 | ATCCACACGTCTCTGCTATC |  |
| p1383 | TGATTACGCCAAGCTGAACGACAGATCGGGATCGC |  |
| p1384 | GCAGGCATGCAAGCTAAACGCACCATCGCTCAGCT |  |
| p1385 | CGCCAAGAAACACGACAAGC |  |
| p1389 | TGATTACGCCAAGCTGGCTGATGAAGCTCTCGTCC |  |
| p1390 | GCAGGCATGCAAGCTATATCACCCGCTGCGAGGAA |  |
| p1391 | CCCGGAAGATCGCTGCAAAG |  |
| p1392 | TGATTACGCCAAGCTGCCGACGGAAGTGACGATGT |  |
| p1393 | GCAGGCATGCAAGCTTTCTTCGAGGACCAGGGCAT |  |
| p1394 | CGTCAGGCGGGCTTTTCTTG |  |
| p1395 | TGATTACGCCAAGCTGCACCCGTTGCGAAGGCCAG |  |
| p1396 | GCAGGCATGCAAGCTCTCATGGCTTTCGTGCAGGG |  |
| p1397 | GTGCTTCTTGTAGTAGTCGG |  |
| p1398 | TGATTACGCCAAGCTACCAGGGAAGCCCAGCACAG |  |
| p1399 | GCAGGCATGCAAGCTGGTGTGATAGTTGACGACCC |  |
| p1400 | AGCGAGGGAAGTGTCGCCAA |  |
| p1401 | TGATTACGCCAAGCTCTGCGGCACCCTTGGAGCTC |  |
| p1402 | GCAGGCATGCAAGCTGCATCGTCGGTTTCCTGACC |  |
| p1403 | CGGCTGAGTGGAACGAGAAC |  |
| p1404 | TGATTACGCCAAGCTCCGACCGCAACATCGCCATG |  |
| p1405 | GCAGGCATGCAAGCTACAAAGACGAATTCCGGGCG |  |
| p1406 | CGGATATTGGAGCGTTCGCC |  |
| p1407 | TGATTACGCCAAGCTCTGCCAAGCCACCTGCCGGA |  |
| p1408 | GCAGGCATGCAAGCTCGATGTGCGATTTCAGCTCC |  |
| p1409 | ATTCGTCCACCCATACCGGC |  |
| p1410 | TGATTACGCCAAGCTGCGCCGCTCAACTCCGTCGA |  |
| p1411 | GCAGGCATGCAAGCTAATAGACCGGGCAGCATGGC |  |
| p1412 | CGTCTGTTGGCTTTCGCTGC |  |
| p1413 | TGATTACGCCAAGCTGCTGCCTCCACGCCATATTC |  |
| p1414 | GCAGGCATGCAAGCTAACGGGTCGAACGAATGTCC |  |
| p1415 | ACGCTCCCGCCATCATGAAG |  |
| p1416 | TGATTACGCCAAGCTGCGACAAGGCGACATCAACT |  |
| p1417 | GCAGGCATGCAAGCTGTGGCGTCGTAAATATCCTC |  |
| p1418 | ATCGGCGGGCATTGTCTCTG |  |
| p1419 | TGATTACGCCAAGCTGCGCCAAGCTGCGGGATGTC |  |
| p1420 | GCAGGCATGCAAGCTAGGAGGTAAAGCGCCTCGGT |  |
| p1421 | AGACCGGCCAGCAGCTTGAT |  |
| p1422 | TGATTACGCCAAGCTGACCGGCTCTGTTCCGCCTT |  |
| p1423 | GCAGGCATGCAAGCTCAGCAAGAGTGAACTGGTCT |  |
| p1424 | ATGTCGTCGAAGCCGATCCG |  |
| p1425 | TGATTACGCCAAGCTGCCGCTGTCGAAAATGTCGC |  |
| p1426 | GCAGGCATGCAAGCTATGTCCTTGGGAACCGGCTT |  |
| p1427 | CATGGGCCGGGATATCGAAT |  |
| p1428 | TGATTACGCCAAGCTCGTTCCACGCGCCGATAAAG |  |
| p1429 | GCAGGCATGCAAGCTATGCAATTCCCGCCGCAATG |  |
| p1430 | ATCTCGTCCCTTGGCAGTTT |  |
| p1431 | TGATTACGCCAAGCTGCCCGAGCTTCTGGTGGATG |  |
| p1432 | GCAGGCATGCAAGCTGCGATGGTTGCACTACTGCA |  |
| p1433 | AGGAGAAGGTGAAGGCGGCA |  |
| p1434 | TGATTACGCCAAGCTCTGCGTCCGTTGCCGACAGG |  |
| p1435 | GCAGGCATGCAAGCTGACCTTATAGCCGTAGCGCC |  |
| p1436 | TCGTGAGCCAGGGAAAGAGA |  |
| p1437 | TGATTACGCCAAGCTGTGCGGAGCACGCTCAGCAG |  |
| p1438 | GCAGGCATGCAAGCTGCCGCGAATATCTGGATGGC |  |
| p1439 | TCCAAGATGTCTCGGCGCTG |  |
| p1440 | TGATTACGCCAAGCTCCTGTCAGATAGATCTCCGG |  |
| p1441 | GCAGGCATGCAAGCTAAAGTAGTCGAGAGCCCTAG |  |
| p1442 | TTCGGCAGAGAGCATCGTCG |  |
| p1443 | TGATTACGCCAAGCTCTTACCGTTCGCCCATGCGC |  |
| p1444 | GCAGGCATGCAAGCTATTGCCGTGCAGGCGGTAGA |  |
| p1445 | GGCGTCATCCAGTGGAAGCA |  |
| p1446 | TGATTACGCCAAGCTGAATGAAATCCCCGCAGCCG |  |
| p1447 | GCAGGCATGCAAGCTGAGCAGCAGCGTCAAAGTAT |  |
| p1448 | TCGGCATTGTAATGATCACG |  |
| p1449 | TGATTACGCCAAGCTGCTTGTTGCCCGTGGAAACC |  |
| p1450 | GCAGGCATGCAAGCTCGATCATGGCAAGTCTCAGT |  |
| p1451 | CCATCTCGGCCACCTGCACT |  |
| p1452 | TGATTACGCCAAGCTGCTGCCTTGCCGCTTCTGTT |  |
| p1453 | GCAGGCATGCAAGCTCAGATCGCCTTGATTGACTT |  |
| p1454 | TGGAGGAGACCGTCACGACA |  |
| p1455 | TGATTACGCCAAGCTCTAGCTGGGAGAGGGTCTTG |  |
| p1456 | GCAGGCATGCAAGCTGGAAGATCTCTTCTACGACA |  |
| p1457 | TCAGGGTCTCGCCCGATCTG |  |
| p1458 | TGATTACGCCAAGCTGGCTTCGGGCCGTCGTCGAA |  |
| p1459 | GCAGGCATGCAAGCTCTCGCGGTTCACTTGGGCCT |  |
| p1460 | TCAGTTGCGGAACGATGGCA |  |
| p1461 | TGATTACGCCAAGCTCCAGCGAGTCAGGCTGAGGT |  |
| p1462 | GCAGGCATGCAAGCTGATCTTGCCGTTGACGTTGG |  |
| p1463 | TGGAGCAGATAAAGGAAGCG |  |
| p1464 | TGATTACGCCAAGCTGAATGCATCGGTATGTTCGC |  |
| p1465 | GCAGGCATGCAAGCTGCAAGGAGACTCTCATGAAA |  |
| p1466 | GCGGGCGTGATGTGAAGGTT |  |
| p1467 | TGATTACGCCAAGCTGGTCCGTTGTTGTTCGGAGC |  |
| p1468 | GCAGGCATGCAAGCTAAAACGCTCACCGGCACATG |  |
| p1469 | TCCAGTTCGCAACTCCGACG |  |
| p1473 | TGATTACGCCAAGCTGGTACCCGCCATCTTTCGCA |  |
| p1474 | GCAGGCATGCAAGCTGACCTGCAAGCGCCTTGAAA |  |
| p1475 | GCGAACTGATCGCCATTCGA |  |
| p1478 | TGATTACGCCAAGCTCGGTGTCCCGCATTGCCACC |  |
| p1479 | GCAGGCATGCAAGCTGCGTGCCGGTTGCGAGAATA |  |
| p1480 | GCAAACCCCGCAAGCAACAC |  |
| p1523 | CGGGTATCCAGATGAATCGG |  |
| p1545 | TGATTACGCCAAGCTCCTGACGCCCGCCGAACACA |  |
| p1546 | GCAGGCATGCAAGCTATGTTCCAGGCATCCCTGCG |  |
| p1547 | GGCGGGTTCCTTTGCGGTAA |  |
| p1548 | TGATTACGCCAAGCTGGCAATCGGTTTAGCCCGGA |  |
| p1549 | GCAGGCATGCAAGCTATGAAGCCCGCGATCAGATA |  |
| p1550 | CAACGAAATGCCAGGCGAGC |  |
| p1551 | TGATTACGCCAAGCTCAGAACGCAGCCTTGCTGCT |  |
| p1552 | GCAGGCATGCAAGCTTTATCGCTCTGGAGCTCGAG |  |
| p1553 | CGTGGTGAATGCCTGCGGTA |  |
| p1557 | TGATTACGCCAAGCTGTCGTTCGATAGGACCCGCC |  |
| p1558 | GCAGGCATGCAAGCTATCCGACGCAGATGAACAGC |  |
| p1559 | TATCCCGTCCTGGCTTTGGC |  |
| p1560 | TGATTACGCCAAGCTGCCAACGACCCGAACCGCCC |  |
| p1561 | GCAGGCATGCAAGCTAGCGCTGTTGTCGGTCGGAG |  |
| p1562 | CGATGAGGCGGCTGAGCTTT |  |
| p1636 | TGATTACGCCAAGCTGGTTGAAAGTGCAGCGACCG |  |
| p1637 | GCAGGCATGCAAGCTCTGTCGACGCAATGGATCGA |  |
| p1638 | TTGTGATCGGCCTTTGTCGG |  |
| p1639 | TGATTACGCCAAGCTGGCATTTGATGCAATGACCG |  |
| p1640 | GCAGGCATGCAAGCTGTGATCTTCGACATCGATTA |  |
| p1641 | CTAAGTCTACTGATGTCCGC |  |
|  |  |  |
| **Primers for qRT-PCR** |  |  |
| p827 | CGCCTGCATGCCGTCGATCC |  |
| p828 | CGCCGCAAATGTCCTGCTCG |  |
| p1476 | CGCCGACCTTGTTGAGAATG |  |
| p1477 | TGACGATGAACCAGGACCTC |  |
| p1594 | CGATGACGAACACGGCTGGA |  |
| p1595 | GTACCGAAACGCTGCGTTGT |  |
| p1596 | ATCCTCATGAAGATTGCCCA |  |
| p1597 | CGAAATAACGGAAATGGTCG |  |
| p1598 | TCGACTACCAGGACGGCATG |  |
| p1599 | CCTGAGATGCCTGTTGCGAG |  |
| p1600 | GGCACGCCTTCCACACCAAA |  |
| p1601 | TGCTTTCCGATACCGCCTGC |  |
| p1604 | TGCCCGCTTCTATCTTCAAC |  |
| p1605 | CCAACAACCATGTCATCGAC |  |
| p1606 | TCTTGGCCGAAAGGTGGAGC |  |
| p1607 | AGGGCGCAATCAGAAGGTCG |  |
| p1608 | AGGGCATTGGCATCACGGAG |  |
| p1609 | AACCACGGGACCGTTCGACA |  |
| p1610 | GCCGTCAGGGCATTGAGGAA |  |
| p1611 | TTGCCGATGGCGACATTCTG |  |
| p1612 | AGGCGGACAACGATTTCAAC |  |
| p1613 | CGAAAGAACGATTGGGTCCT |  |
| p1614 | AGACGAAATTATAGGCGAGC |  |
| p1615 | AACCTGTTGCCAATGTCTTC |  |
| p1616 | CGAAGCCGCCATCAAGAAGG |  |
| p1617 | CCTGCGAGATCAGCGATTCG |  |
| p1618 | ACGGCGAATCGTTCCAGGAG |  |
| p1619 | TGACCTGGGTTACGCCTTGG |  |
| p1620 | TACCGCAATACGCTACTTTG |  |
| p1621 | AACGGCTATGAGAATGACAG |  |
| p1622 | TATGCCTCAGCGACGGATGG |  |
| p1623 | GCTGGCATCGGTTCGGAAGA |  |
| p1624 | CGCTATTCCGCTGGTGCTCA |  |
| p1625 | CGCCGATATAGGTGCCTGCA |  |
| p1626 | GGTCACATCCATCATCAGGC |  |
| p1627 | GTTGATCAGGATGTCGACCG |  |
| p1634 | ACACTCTGGAAGGCTGGAAG |  |
| p1635 | AGAAGCGGAAGCAGGTCTTC |  |

Figurski, D.H., and Helinski, D.R. (1979) Replication of an origin-containing derivative of plasmid RK2 dependent on a plasmid function provided in trans. *Proc Natl Acad Sci USA* 76: 1648-1652.

Johnston, A.W.B., and Beringer, J.E. (1975) Identification of the *Rhizobium* strains in pea root nodules using genetic markers. *Journal of General Microbiology* 87: 343-350.

Schafer, A., Tauch, A., Jager, W., Kalinowski, J., Thierbach, G., and Puhler, A. (1994) Small mobilizable multipurpose cloning vectors derived from the *Escherichia coli* plasmids pK18 and pK19 - selection of defined deletions in the chromosome of *Corynebacterium glutamicum*. *Gene* 145: 69-73.
