# Supplementary material for: Adaptation of Rhizobium leguminosarum to pea, alfalfa and sugar beet rhizospheres investigated by comparative transcriptomics
Source: Genome Biol. 2011 Oct 21;12(10):R106. doi: 10.1186/gb-2011-12-10-r106 (PMC3333776; doi:10.1186/gb-2011-12-10-r106)
Supplement: Additional file 7 — Table S2 - genes whose expression was differentially regulated by three-fold or more in the rhizospheres of pea, alfalfa and sugar beet at 7 dpi of 7-day-old plants compared to free-living Rlv3841. In addition, genes specifically up-regulated in the pea rhizosphere are shown compared to free-living cells and those in the alfalfa rhizosphere and the sugar beet rhizosphere. [file gb-2011-12-10-r106-S7.DOC]

**Table S7. Real time-quantitative reverse transcription PCR and log fold values calculated by comparative ct method.**

| **Gene ID** | **Gene Name** | **qRT-PCR**  **Log_2_fold +/-SEM** | **Microarray Log_2_fold** |
| --- | --- | --- | --- |
| **Up-regulated genes** | | | |
| RL4267 | putative acetaldehyde dehydrogenase | 6.34 +/- 1.49 | 9.95 |
| pRL100169 | rhizosphere expressed protein RhiA | 4.28 +/- 0.72 | 9.52 |
| RL3424 | putative C4-dicarboxylate transport protein | 6.47 +/- 0.36 | 9.47 |
| RL1251 | putative serine protease | 4.49 +/- 0.49 | 8.29 |
| RL1860 | putative phenylalanine-4-hydroxylase | 6.80 +/- 0.75 | 7.06 |
| RL0996 | putative transmembrane transporter | 3.84 +/- 0.71 | 6.04 |
| RL0037 | putative phosphoenolpyruvate carboxykinase | 4.25 +/- 0.48 | 5.93 |
| pRL80026 | putative branched-chain amino acid ABC transporter binding component | 5.06 +/- 0.68 | 4.61 |
| RL4274 | putative HlyD family transmembrane efflux protein | 5.64 +/- 1.29 | 4.46 |
| pRL100187 | N-acetylglucosaminyltransferase | 4.57 +/- 0.36 | 4.04 |
| RL1925 | conserved hypothetical protein | 5.26 +/- 0.18 | 3.96 |
| pRL110443 | putative hydroxyethylthiazole kinase | 1.55 +/- 1.37 | 2.45 |
| **Down-regulated genes** | | | |
| RL2164 | hypothetical protein | -2.16 +/- 0.17 | -3.53 |
| pRL100451 | putative autoaggregation protein | -3.66 +/- 0.58 | -4.79 |
| RL3624 | putative substrate-binding component of ABC transporter | -4.63 +/- 0.9 | -8.61 |
| **House-keeping gene** | | | |
| RL0644 | putative ribitol 2-dehydrogenase | 0 | 0 |
