# Supplementary material for: Adaptation of Rhizobium leguminosarum to pea, alfalfa and sugar beet rhizospheres investigated by comparative transcriptomics
Source: Genome Biol. 2011 Oct 21;12(10):R106. doi: 10.1186/gb-2011-12-10-r106 (PMC3333776; doi:10.1186/gb-2011-12-10-r106)
Supplement: Additional file 8 — Table S4 - data used to draw Figures 1, 2 and 3and results for competition in pea and alfalfa rhizospheres of mutants compared with Rlv3841. [file gb-2011-12-10-r106-S8.DOC]

46

187

3d

(269)

7d

(303)

13

13

18

18

50

20

48

1d

(273)

**UP-REGULATED IN PEA RHIZOSPHERE AFTER DIFFERENT LENGTH OF INCUBATION**

**(387)**

**A (i)**

**A (ii)**

77

257

3d

(413)

7d

(461)

13

13

26

53

96

31

51

1d

(365)

**DOWN-REGULATED**

**IN PEA RHIZOSPHERE AFTER DIFFERENT LENGTH OF INCUBATION**

**(591)**

**Fig. S2. Effect of length of incubation in the pea rhizosphere; Venn diagrams of Rlv3841 genes differentially regulated in the pea rhizosphere at 1, 3 and 7 dpi of 7d-old plants (A) up-regulated and (B) down-regulated.** Total genes differentially regulated in each rhizosphere are shown in brackets. Venn diagrams were drawn in GeneSpring by selecting differentially regulated genes (≥3-fold, filtered on confidence p≤0.05) for each condition.

A
