# Supplementary material for: Adaptation of Rhizobium leguminosarum to pea, alfalfa and sugar beet rhizospheres investigated by comparative transcriptomics
Source: Genome Biol. 2011 Oct 21;12(10):R106. doi: 10.1186/gb-2011-12-10-r106 (PMC3333776; doi:10.1186/gb-2011-12-10-r106)
Supplement: Additional file 9 — Table S3 - genes whose expression was differentially regulated by three-fold or more compared to free-living Rlv3841 when grown in the presence of formate, protocatechuate, 4-hydroxybenzoate, phenylalanine, proline, L-arabinose, galactose, arabinogalactan and under conditions of N-limitation. [file gb-2011-12-10-r106-S9.DOC]

39

106

alfalfa

(299)

SB

(186)

13

13

52

102

28

13

112

pea

(283)

**UP-REGULATED**

**(768)**

**A (i)**

**A (ii)**

29

184

alfalfa

(343)

SB

(343)

13

13

58

72

46

84

149

pea

(475)

**DOWN-REGULATED**

**(1161)**

**B (i)**

↑alfalfa v pea (7)

↑SB v pea (6)

↑SB v alfalfa (3)

alfalfa-SB

rhizospheres

comparison

pea-SB rhizospheres

comparison

↑alfalfa v SB (14)

19

30

5

12

2

6

3

0

pea-alfalfa

rhizospheres

comparison

↑pea v SB (49)

↑pea v alfalfa (43)

13

A

↑pea v SB (49)

↑alfalfa v SB (14)

40

5

9

**B (ii)**

**Fig. S1. Venn diagrams of Rlv3841 genes differentially regulated (≥3-fold, p≤0.05**) **in (A) an indirect comparison of rhizospheres in which pea, alfalfa and sugar beet rhizospheres are investigated using comparison with glucose-grown free-living cells (i) up-regulated and (ii) down-regulated, (B) up-regulated in direct comparison of (i) pea, alfalfa and sugar beet rhizospheres and (ii) legume and sugar beet rhizospheres.** Total genes differentially regulated in each rhizosphere are shown in brackets and listed for A (i) and (ii) in Table S2, ii-iv & vii-ix. Of the 106 genes up-regulated by all three plant rhizospheres (A (i), Table S2, i), 66% are annotated as hypothetical and 15% are membrane proteins or concerned with cell surface. The 184 genes down-regulated in all rhizospheres (A (ii), Table S2, vi) include those for proteins involved in general cellular functions of bacterial motility and chemotaxis (6%), tRNA and DNA synthesis, chromosome and plasmid replication, cell division, protein export by the Sec system, electron transport and formation of ATP. In addition, 12% are annotated as hypothetical, 13% are membrane proteins or concerned with cell surface and 16% are ribosomal proteins. Components of two ABC transport systems which import glucose (RL3624-5, RL4252) (Karunakaran *et al.,* 2009) are down-regulated in all rhizospheres in comparison with glucose-grown cells (Table S2, vi). From direct comparison of the pea rhizosphere with those of alfalfa and sugar beet (B (i)) there are 30 pea rhizosphere-specific genes (listed in Table S2, xi). There are 9 legume rhizosphere-specific genes, up-regulated in both the pea and alfalfa rhizosphere compared to that of sugar beet (B (ii)). Abbreviations: SB, sugar beet.
