# Supplementary material for: Adaptation of Rhizobium leguminosarum to pea, alfalfa and sugar beet rhizospheres investigated by comparative transcriptomics
Source: Genome Biol. 2011 Oct 21;12(10):R106. doi: 10.1186/gb-2011-12-10-r106 (PMC3333776; doi:10.1186/gb-2011-12-10-r106)
Supplement: Additional file 10 — Table S5 - identification of putative substrates for ABC, TRAP and MFS transporters of R. leguminosarum 3841. [file gb-2011-12-10-r106-S10.DOC]

**Table S5. Identification of putative substrates for ABC, TRAP and MFS transporters of *R. leguminosarum* 3841**

| **Gene** | **Transporter** | **Putative substrate** | **Evidence** | **Reference** |
| --- | --- | --- | --- | --- |
| pRL80060 | ABC PAAT | Mimosine-like | 44-79% id to system importing toxic alkaloid mimosine in the mimosa-nodulating *Rhizobium* sp. TAL1145. | (Borthak*ur et a*l., 2003) |
| pRL100248 | ABC POPT | GABA-like | GABA transport system. | (Whi*te et a*l., 2009) |
| pRL110243 | ABC PepT | α galactoside | Pairs with SMb21647 (Agp) (75% id) induced by melibiose/raffinose/galactose. | (Mauchli*ne et a*l., 2006, Gage & Long, 1998) |
| pRL110281 | ABC PepT | Arabinan | Neighbouring genes involved in arabinan breakdown. |  |
| pRL110400-4* | ABC HAAT | Acetoacetate | Elevated on microarray with added acetoacetate in *R. leguminosarum* 3841. | (Karunakar*an et a*l., 2009) |
| pRL120500 | TRAP | Protocatechuate | Elevated on microarray with added protocatechuate in *R. leguminosarum* 3841. | This work |
| RL0996 | MFS | Tartrate | 87% id to tartrate transporter protein of *Agrobacterium radiobacter* K84  Contiguous with tartrate dehydrogenase (RL0995). | (Slat*er et a*l., 2009) |
| RL2377-8* | ABC CUT2 | Arabinose | Elevated on microarrays with added L-arabinose in *R. leguminosarum* 3841. | This work |
| RL2720 | ABC CUT2 | Arabinogalactan | Conserved domain: AraH-like for uptake of arabinose (*E.coli*), similar are domains for uptake of ribose, allose and galactose  Elevated on microarray with added arabinogalactan in *R. leguminosarum* 3841.  Not elevated in microarrays grown on L-arabinose or galactose in *R. leguminosarum* 3841. | This work  This work |
| RL3040 | ABC MolT | Molybdate | An orphan SBP (no other components encoded in close proximity) of MolT family; 80% id to probable molybdate SBP *R. etli* CF42 BUT only 27% id to RL4685, the only other SBP of MolT system (it is the closest SBP though!) which is induced by molybdate limitation. | (Mauchlin*e et* al., 2006) |
| RL3615-6 * | ABC CUT2 | Arabinose | Elevated on microarrays with added L-arabinose in *R. leguminosarum* 3841. | This work |
| RL3721 | ABC NitT | Uracil/uridine | SBP RL3723 pairs with SMc01827 (69% id), a system induced by uracil and uridine. | (Mauchlin*e et* al., 2006) |
| RL3840 | ABC CUT2 | Melibiose/raffinose/lactose | Pairs with SMb20931 (91% id) induced by raffinose, melibiose, lactose. | (Mauchlin*e et a*l., 2006) |
| RL3906-10* | ABC HAAT | 4-hydroxybutyrate | Highly elevated on microarrays with added 4-hydroxybutyrate in *R. leguminosarum* 3841.  SBP RL3906 shows 88% id to SMb20568 of *S. meliloti* whose expression was induced by both 4-hydroxybenzoate and protocatechuate. | This work  (Mauchlin*e et* al., 2006) |
| RL4218 | ABC CUT1 | Sorbitol/mannitol/dulcitol | Shows 60% id with MtlE of *Pseudomonas fluorescens* and 87% id with SMc01496 from  *S. meliloti* (described as ‘probable sorbitol-binding periplasmic protein’)  SMc01496 is induced by dulcitol, sorbitol, mannitol. | (Mauchlin*e et* al., 2006) |
| RL4655 | ABC CUT2 | *myo*-inositol | *myo-*inositol uptake system.  Elevated on microarray with added *myo-*inositol in *R. leguminosarum* 3841. | (Karunakara*n et a*l., 2009, F*ry et a*l., 2001) |
| RL4709 | MFS | Shikimate | Shikimate is taken up by an MFS transporter showing 79% id to NGR_c33200 from *Rhizobium* sp. NGR234. | (Schmeiss*er et a*l., 2009) |

* Not elevated in any rhizosphere tested.

Borthakur, D., M. Soedarjo, P. M. Fox & D. T. Webb, (2003) The *mid* genes of *Rhizobium* sp strain TAL1145 are required for degradation of mimosine into 3-hydroxy-4-pyridone and are inducible by mimosine. *Microbiol.* **149**: 537-546.

Fry, J., M. Wood & P. S. Poole, (2001) Investigation of myo-inositol catabolism in *Rhizobium leguminosarum* bv. *viciae* and its effect on nodulation competitiveness. *Mol. Plant-Microbe Interact.* **14**: 1016-1025.

Gage, D. J. & S. R. Long, (1998) a-Galactoside uptake in *Rhizobium meliloti*: Isolation and characterization of *agpA*, a gene encoding a periplasmic binding protein required for melibiose and raffinose utilization. *J. Bacteriol.* **180**: 5739-5748.

Karunakaran, R., V. K. Ramachandran, J. C. Seaman, A. K. East, B. Moushine, T. H. Mauchline, J. Prell, A. Skeffington & P. S. Poole, (2009) Transcriptomic analysis of *Rhizobium leguminosarum* b.v. *viciae* in symbiosis with host plants *Pisum sativum* and *Vicia cracca*. *J. Bacteriol.* **191**: 4002-4014.

Mauchline, T. H., J. E. Fowler, A. K. East, A. L. Sartor, R. Zaheer, A. H. F. Hosie, P. S. Poole & T. M. Finan, (2006) Mapping the *Sinorhizobium meliloti* 1021 solute-binding protein-dependent transportome. *Proc. Natl. Acad. Sci. U.S.A* **103**: 17933-17938.

Schmeisser, C., H. Liesegang, D. Krysciak, N. Bakkou, A. Le Quere, A. Wollherr, I. Heinemeyer, B. Morgenstern, A. Pommerening-Roser, M. Flores, R. Palacios, S. Brenner, G. Gottschalk, R. A. Schmitz, W. J. Broughton, X. Perret, A. W. Strittmatter & W. R. Streit, (2009) Rhizobium sp strain NGR234 possesses a remarkable number of secretion systems. *Appl. Environ. Microbiol.* **75**: 4035-4045.

Slater, S. C., B. S. Goldman, B. Goodner, J. C. Setubal, S. K. Farrand, E. W. Nester, T. J. Burr, L. Banta, A. W. Dickerman, I. Paulsen, L. Otten, G. Suen, R. Welch, N. F. Almeida, F. Arnold, O. T. Burton, Z. J. Du, A. Ewing, E. Godsy, S. Heisel, K. L. Houmiel, J. Jhaveri, J. Lu, N. M. Miller, S. Norton, Q. Chen, W. Phoolcharoen, V. Ohlin, D. Ondrusek, N. Pride, S. L. Stricklin, J. Sun, C. Wheeler, L. Wilson, H. J. Zhu & D. W. Wood, (2009) Genome Sequences of Three Agrobacterium Biovars Help Elucidate the Evolution of Multichromosome Genomes in Bacteria. *J. Bacteriol.* **191**: 2501-2511.

White, J. P., J. Prell, V. K. Ramachandran & P. S. Poole, (2009) Characterization of a gamma-aminobutyric acid transport system of *Rhizobium leguminosarum* bv. *viciae* 3841. *J. Bacteriol.* **191**: 1547-1555.
